# Change in Right-to-Left Shunt Fraction in Patients with Chronic Thromboembolic Pulmonary Hypertension after Pulmonary Endarterectomy

**DOI:** 10.3390/jcdd10110442

**Published:** 2023-10-25

**Authors:** Lena Reimann, Laura Mayer, Simon Raphael Schneider, Esther I. Schwarz, Julian Müller, Anna Titz, Michael Furian, Arcangelo F. Carta, Harry Etienne, Bianca Battilana, Stéphanie Saxer, Thomas Pfammatter, Thomas Frauenfelder, Isabelle Opitz, Silvia Ulrich, Mona Lichtblau

**Affiliations:** 1 Department of Pulmonology, University Hospital Zurich, University of Zurich, 8091 Zurich, Switzerland; 2 Department of Thoracic Surgery, University Hospital Zurich, 8091 Zurich, Switzerland; 3 Department of Health, Eastern Switzerland University of Applied Sciences, 9001 St. Gallen, Switzerland; 4 Institute of Diagnostic and Interventional Radiology, University Hospital Zurich, University of Zurich, 8091 Zurich, Switzerland

**Keywords:** pulmonary hypertension, chronic thromboembolic pulmonary hypertension, right-to-left shunt, hyperoxia, right heart catheterization, pulmonary endarterectomy, risk stratification

## Abstract

Background: Pulmonary endarterectomy (PEA) is the treatment of choice for patients with chronic thromboembolic pulmonary hypertension (CTEPH) with accessible lesions. Breathing pure oxygen (hyperoxia) during right heart catheterization (RHC) allows for the calculation of the right-to-left shunt fraction (Qs/Qt). In the absence of intracardiac shunt, Qs/Qt can be used as a marker of ventilation–perfusion mismatch in patients with CTEPH. This study involved investigating Qs/Qt after PEA and its relation to other disease-specific outcomes. Study design and Methods: This study is a retrospective study that focuses on patients with operable CTEPH who had Qs/Qt assessment during RHC before and 1 year after PEA. Additionally, 6 min walking distance (6MWD), WHO functional class (WHO-FC), and NT-proBNP were assessed to calculate a four-strata risk score. Results: Overall, 16 patients (6 females) with a median age of 66 years (quartiles 55; 74) were included. After PEA, an improvement in mean pulmonary artery pressure (38 [32; 41] to 24 [18; 28] mmHg), pulmonary vascular resistance (5.7 [4.0; 6.8] to 2.5 [1.4; 3.8] WU), oxygen saturation (92 [88; 93]% to 94 [93; 95]%), WHO-FC, and risk score was observed (all *p* < 0.05). No improvement in median Qs/Qt could be detected (13.7 [10.0; 17.5]% to 13.0 [11.2; 15.6]%, *p* = 0.679). A total of 7 patients with improved Qs/Qt had a significant reduction in risk score compared to those without improved Qs/Qt. Conclusion: PEA did not alter Qs/Qt assessed after 1 year in operable CTEPH despite an improvement in hemodynamics and risk score, potentially indicating a persistent microvasculopathy. In patients whose shunt fraction improved with PEA, the reduced shunt was associated with an improvement in risk score.

## 1. Introduction

Precapillary pulmonary hypertension (PH) is defined as mean pulmonary artery pressure (mPAP) > 20 mmHg, pulmonary artery wedge pressure (PAWP) ≤ 15 mmHg, and pulmonary vascular resistance (PVR) > 2 WU (assessed via right heart catheterization) [1]. Based on its different pathophysiological mechanisms, clinical presentations, and hemodynamic characteristics, PH can be divided into five main groups [1,2,3]. The incidence and prevalence of patients diagnosed with group 4 PH or chronic thromboembolic PH (CTEPH) is 2–6 and 26–38 cases/million adults, with the increasing number over recent years potentially being related to improved diagnostics [1]. Typical symptoms include exertional dyspnea, along with impaired exercise performance and reduced quality of life [4,5,6]. Due to pathophysiological changes such as fibrotic scars in more proximal pulmonary arteries and vascular remodeling or intimal thickening (known as distal microvasculopathy), pulmonary vascular resistance (PVR) increases, resulting in an elevated pulmonary artery pressure (PAP) [7,8]. Increased ventilatory drive leads to inefficient ventilation. Impaired cardiac output and elevated ventilation–perfusion mismatch with the right–left shunt result in arterial and mixed venous hypoxemia (also caused by reduced pulmonary diffusion capacity) [6,9]. The current treatment of CTEPH depends on the location of the lesions and involves combinations of pulmonary endarterectomy (PEA), balloon pulmonary angioplasty (BPA), targeted medication, and general measures such as exercise training and anticoagulation [1]. According to the current ERS/ESC guidelines [1], PEA, the surgical removal of thromboembolic material from the pulmonary vessels is considered the treatment of choice to reach normal pulmonary hemodynamics in patients with accessible lesions [10].

As shown in a recently published study, a high proportion of patients with precapillary PH due to pulmonary vascular disease (PAH/CTEPH) showed an increased fraction of right-to-left shunt (Qs/Qt) (measured by RHC) [11]. Right-to-left shunts occur when blood flows from the right to the left side of the heart without first being oxygenated by passing through the pulmonary capillaries. In the absence of an intracardiac right-to-left shunt, these intrapulmonary shunts can arise due to physiological reasons such as the perfusion of non-ventilated alveoli and pulmonary arterio-venous shunts. The inhalation of pure oxygen (hyperoxia, FiO_2_ 1.0) during RHC allows for the calculation of Qs/Qt [11]. Data on the effect of PEA on shunt fraction are scant.

The purpose of this study was to investigate the change in the right-to-left shunt fraction after PEA (assessed during RHC). In addition, we aimed to compare the improvement in shunt fraction to hemodynamic parameters and other outcomes, such as changes in 6 min walking distance (6MWD), WHO functional class, levels of NT-pro brain natriuretic peptide (NT-proBNP), and, consequently, the possible changes in risk category with respect to the four-strata risk assessment tool.

## 2. Materials and Methods

### 2.1. Study Design and Participants

In this retrospective study, we examined Qs/Qt and other hemodynamics assessed during the clinically indicated RHC of patients with CTEPH before and 1 year after PEA. All RHCs were performed at the PH-Center, University Hospital Zurich, Switzerland [11]. This study’s sample included patients ≥18 years of both sexes and with PH (according to current ESC/ERS Guidelines [1]—defined as mPAP > 20 mmHg, PAWP ≤ 15 mmHg, and PVR > 2.0 WU), as well as the diagnosis of CTEPH according to a thorough investigation, including pulmonary angiography, a ventilation–perfusion scan, and other required measures [12]. Patients belonging to PH groups other than CTEPH were excluded. Each patient underwent a RHC before and 1 year after PEA during the period of March 2016 to April 2022. Only patients that had undergone measurements of hemodynamic parameters while breathing ambient air (normoxia, FiO_2_ 0.21) and pure oxygen (hyperoxia, FiO_2_ 1.0) for 10 min each were included in the analysis. All patients provided written informed consent. The study protocol was approved by the Cantonal Ethics Committee Zurich (No. 2020-01541).

### 2.2. Measurements

The RHC was performed via jugular venous access using a balloon-tipped, triple-lumen, fluid-filled, 8 French Swan-Ganz CCOmbo V catheter (Edwards Lifesciences, Irvine, CA, USA) in supine resting position following a previously described method [7]. Zero leveling was performed by placing transducers that were adapted to the atmospheric pressure at mid-chest level (as an approximation of the left atrium) [13,14]. Baseline measurements were collected whilst the patients were breathing ambient air (FiO_2_ 0.21), and additional measurements were taken whilst patients were breathing pure oxygen (hyperoxia, FiO_2_ 1.0) via a three-way non-rebreathing valve from a reservoir bag (AmbuSPUR-II, Synmedic AG, Zurich, Switzerland) for >10 min. The hemodynamic parameters, heart rate (HR), systolic, mean and diastolic systemic blood pressure (sSBP, mSBP, dSBP), systolic, mean and diastolic PAP (sPAP, mPAP, dPAP), pulmonary arterial wedge pressure (PAWP), and right atrial pressure were averaged over several respiratory cycles at baseline and whilst breathing hyperoxic gas for >10 min [7,11,13]. At both measurement time points, blood samples were taken from the pulmonary artery catheter tips and via radial artery puncture to assess hemoglobin (Hb), lactate, arterial and mixed venous oxygen saturation (SaO_2_, SmvO_2_), arterial and mixed venous partial pressures of oxygen (PaO_2_, PmvO_2_), and carbon dioxide (PaCO_2_, PmvO_2_) [7,11]. Cardiac output (CO) was determined using thermodilution (Edwards Lifesciences, Irvine, CA, USA) [11]. Through further using body surface area (BSA), the cardiac index (CI) was calculated as CO/BSA [7]. The right-to-left shunt fraction was computed from measurements taken during pure oxygen breathing using the formula described by Chiang et al. [15]:

$$\frac{Qs}{Qt}=\frac{\left(\left(0.0031*\left({FiO}_{2}*\left(P_{B}-P_{H2O}-{PaCO}_{2}/R\right)\right)\right)+\left({ScapO}_{2}*Hb*1.34\right)\right)-\left(\left(1.34*Hb*{SaO}_{2}\right)+\left(0.0031*{PaO}_{2}\right)\right)}{\left(\left(0.0031*\left({FiO}_{2}*\left(P_{B}-P_{H2O}-{PaCO}_{2}/R\right)\right)\right)+\left({ScapO}_{2}*Hb*1.34\right)\right)-\left(\left(1.34*Hb*{SmvO}_{2}\right)+\left(0.0031*{PmvO}_{2}\right)\right)}$$

*FiO_2_*—fraction of inspired oxygen; *P_B_*—standard barometric pressure; *P_H2O_*—partial pressure of water at body temperature; *PaCO_2_*—arterial partial pressure of carbon dioxide; *R*—respiratory quotient; *ScapO_2_*—pulmonary capillary oxygen saturation; *Hb*—hemoglobin; *SaO_2_*—arterial saturation of oxygen; *PaO_2_*—arterial partial pressure of oxygen; *SmvO_2_*—mixed venous saturation of oxygen; *PmvO_2_*—mixed venous partial pressure.

6MWD, WHO functional class, and NT-proBNP levels were evaluated shortly before pulmonary endarterectomy and during the 1-year post-surgical follow-up. The collected variables were used to calculate the risk score by using the simplified four-strata risk assessment tool as recommended to determine 1-year mortality in patients with pulmonary arterial hypertension [16].

### 2.3. Outcomes

The main outcomes were the change in Qs/Qt 1 year after PEA and the association between Qs/Qt changes and the changes in the hemodynamic parameters (mPAP, PVR, PAWP, and CI). Other outcomes included changes in WHO functional class, 6MWD, NT-proBNP levels, and change in four-strata risk category (assessed by risk score) compared to the improvement in shunt fraction.

### 2.4. Statistics

Categorical data are reported as number (percentage), and continuous data are reported as median (interquartile range). Due to the small sample size of 16 patients nonparametric tests were used. The Wilcoxon signed-rank test was used to compare the parameters measured before and after PEA. After forming two groups by virtue of a decreased shunt fraction, which was considered as an improvement in Qs/Qt of >10% compared to baseline and no improvement, parameters such as hemodynamics, 6MWD, NT-proBNP, risk score and corresponding four-strata risk category, and WHO functional class were compared before and after PEA for each group separately by using the Wilcoxon signed-rank test. The Mann–Whitney U test was used to compare patients with and without improvements in Qs/Qt. Additionally, Fisher’s exact test was used where appropriate to analyze changes in WHO functional class, 6MWD, NT-proBNP levels, and the risk scores or four-strata risk categories related to the shunt groups. For all tests, a *p*-value <0.05 was considered statistically significant. Missing values were not replaced. The statistical analyses were performed using IBM SPSS Statistics (version 28.0.1.1, SSPS Chicago, IL, USA).

## 3. Results

### 3.1. Study Population

**Figure 1:**
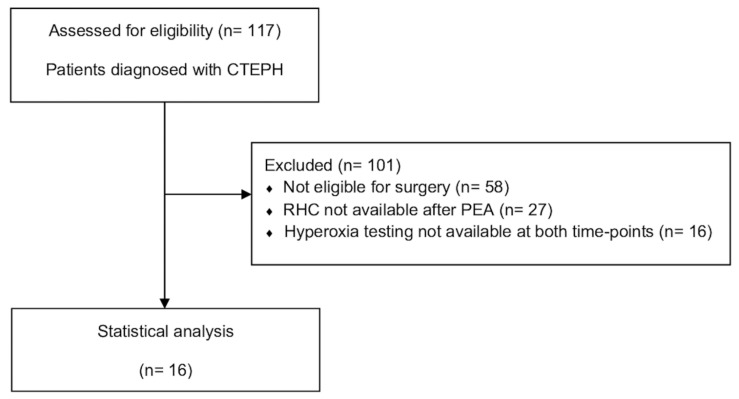
Patient flow. CTEPH—chronic thromboembolic pulmonary hypertension; PEA—pulmonary endarterectomy; RHC—right heart catheterization.

We included 16 patients with CTEPH (38% women); the patients had a median age of 66 (55; 74) years, and Qs/Qt measurements were taken before and 1 year after PEA (Table 1). A flowchart depicting the present study’s patient flow is presented in Figure 1, and the baseline characteristics of the included patients are shown in Table 1. Initial RHC was followed by PEA after 135 (71; 180) days, whereas the median follow-up was 365 (345; 377) days after PEA. Half of the patients were medically pretreated with PH-targeted drugs after hemodynamic evaluation and before undergoing PEA.

Hemodynamics, blood oxygenation, and shunt fraction variables were assessed before and 1 year after pulmonary endarterectomy.

The right-to-left shunt fraction was not different after PEA 13.0 (11.2; 15.6) % compared to before 13.7 (10.0; 17.5) % (*p*-value = 0.679). Fourteen patients (88%) had a shunt fraction >10% after the surgery compared with twelve patients (75%) before PEA (Table 2). Hemodynamic and blood oxygenation variables are shown in Table 3 and Table A1. One year after PEA, pulmonary hemodynamics significantly improved, median mPAP decreased by 14 mmHg, and PVR decreased by 3.2 WU (all *p* < 0.001), whereas no significant changes in PAWP and CI were detected. Furthermore, the arterial and mixed venous blood gas samples showed a significant improvement in oxygen saturation (91 [90; 95]% to 97 [95; 98]%, *p* = 0.001), PaO_2_ (8.5 [6.9; 9.3] to 10.2 [9.4; 11.0] kPa, *p* = 0.002), and PaCO_2_ (4.4 [3.9; 4.7] to 4.6 [4.3; 5.2] kPa, *p* = 0.034) after the surgical procedure (Table 3). Additionally, WHO functional class and the four-strata risk score improved significantly (Table 4). On the contrary, there was no significant change in exercise performance (measured via 6 min walking distance and oxygen saturation at end of exercise or determined NT-proBNP levels).

### 3.2. Comparison of Patients with and without Improvement in Shunt Fraction

In both groups, there was a significant improvement in mPAP, PVR, and WHO functional class one year after PEA, whereas 6MWD and NT-proBNP remained unchanged. Only patients with improved shunt fraction, which was considered as an improvement in Qs/Qt > 10% compared to baseline, significantly improved their risk score (Table 5). Half of the patients showed a residual/persistent PH after PEA, and change in shunt fraction was not predictive for the appearance of residual PH (Table 6).

## 4. Discussion

In this retrospective study, we investigated the right-to-left shunt fraction (Qs/Qt) in patients with CTEPH whilst breathing pure oxygen (FiO_2_ 1.0) during RHC at baseline and one year after PEA. Among all patients, the median shunt fraction was unchanged 1 year after PEA, but 7/16 (44%) patients improved their shunt fraction (>10% from baseline). A year after PEA, mPAP, PVR, oxygen saturation, and PaO_2_ on ambient air were significantly improved, and WHO-FC and four-strata risk score also improved, while 6MWD and NT-proBNP remained unaltered. Half of the patients were free of resting PH 1 year after PEA. Regarding the groups stratified by improvement in shunt fraction, both groups showed the same significant hemodynamic improvements as mentioned. The only significant difference between the groups was the decrease in the four-strata risk score in the group with improved Qs/Qt, whereas the group without any improvements in shunt fraction maintained the same risk score one year after PEA, although the absolute risk scores were very low in both groups.

Until now, clinical data related to the right-to-left shunt fraction in pre- and postoperative comparison of PEA were lacking. Conversely, improvements in hemodynamic parameters after PEA have been described several times in the literature [17,18]. Ventilation–perfusion ratio (measured via the multiple inert gas elimination technique) improved significantly in nine patients after PEA [19]. Another study compared Qs/Qt before and after balloon pulmonary angioplasty (BPA), and in line with our findings, neither revealed significant improvements in shunt fraction [20].

A right-to-left shunt fraction of ≤5% is considered physiological, even though the threshold of ≤10% has been used more frequently [11,21]. In our study, we measured a Qs/Qt > 5% in all patients during both measurements. Using the threshold of 10%, 88% of the patients still showed shunt fractions above that threshold 1 year after PEA. Our results demonstrate no significant improvement in Qs/Qt after PEA in the overall population despite significant improvements in hemodynamics, WHO-FC class, and risk score. The lack of improvement in exercise performance can be attributed to the already high 6MWD pre-PEA and the well-documented ceiling effect of 6MWD [22].

Several possible hypotheses might explain our findings regarding the lack of improvement in mean Qs/Qt: First, it is known that a high proportion of patients with CTEPH reveal a microvascular involvement of the disease, which is not accessible to surgery and thus precludes improvement of Qs/Qt with PEA. This microvasculopathy, even if it does not clinically manifest at rest, may be responsible for an increased Qs/Qt [20]. Second, the known component of inflammation involved in the pathogenesis of CTEPH could lead to impaired oxygenation in the lungs [20]. Therefore, a lack of improvement in Qs/Qt might be an indicator of the microvascular involvement of the disease and might help in decision making with respect to post-operative medical PH-targeted therapy. Additionally, Frantz et al. showed a significant dysregulation in vascular tone during vasodilator testing in patients with CTEPH, which could also be a reason for the lack of improvement in Qs/Qt in our study [23]. Similarly, in patients with pulmonary arterial hypertension, as previously described, no decrease in Qs/Qt was observed with medical treatment despite improvements in the hemodynamic parameters [24].

Although oxygen saturation was significantly improved at rest, no improvement in SpO_2_ at peak exercise during 6MWD was demonstrated, potentially indicating persistent microvascular remodeling in these patients, which possibly leads to exercise-induced hemodynamic impairment and might reflect the persistently increased Qs/Qt. However, in a previous study, a high Qs/Qt as a predictor of reduced exercise capacity after BPA could not be reproduced in patients with a history of PEA [25].

Both patient groups that were stratified by shunt improvement revealed similar improvements in hemodynamics after PEA, and no correlation between change in Qs/Qt and improvement in hemodynamics was found. Also, an improvement in oxygen saturation and WHO functional class after PEA was detectable in both groups irrespective of shunt improvement. The substantial difference between the two groups stratified via shunt decrease was a significant reduction in risk score, which was only observed in the group with improved shunt fractions. Previous studies have reported the validation of the three-strata risk assessment tool for patients with CTEPH [26,27], but studies validating the use of the simplified four-strata risk assessment tool (used in this study) in the context of CTEPH are still lacking. Eighty-one percent of the patients already had a low or low intermediate risk score at baseline, which makes it more challenging to demonstrate significant improvements.

Despite the overall improvement one year after PEA, 50% of patients persistently fulfilled the hemodynamic definition of PH according to the current ESC/ERS guidelines [1], which utilize a lower adjusted threshold, with a mPAP > 20 mmHg and PVR > 2 WU. Even when using this lower threshold to facilitate comparisons with earlier studies, this proportion of patients with persistent/residual CTEPH is in line with the findings of previous studies [28,29,30]. In summary, a lack of improvement in shunt fraction after PEA could be used as an indicator for earlier work-up for residual PH after PEA. Shunt fraction measurements (without right heart catheterization) can easily and inexpensively be taken in a clinical setting, and the equipment needed for this is widely accessible. Future prospective trials will need to assess the predictive value of shunt measurements.

## 5. Contribution to the Field Statement

Chronic thromboembolic pulmonary hypertension (CTEPH) is a pulmonary vascular disease characterized by recurrent pulmonary embolisms and the fact that it often follows vascular remodeling, leading to an increase in pulmonary pressure (measured via right heart catheterization). CTEPH affects up to 38 individuals per million adults worldwide. Pulmonary endarterectomy (PEA), the surgical removal of thromboembolic material from the pulmonary vessels, is the current treatment of choice to reach normal pulmonary hemodynamics in operable cases. As already shown in previous studies, many patients with CTEPH show an increased fraction of right-to-left shunt (measured by right heart catheterization). Data on the effect of PEA on shunt fractions are rare. The aim of this study was to investigate the change in the right-to-left shunt fraction after PEA and its relation to other disease-specific outcomes. In this study there was no significant improvement in shunt fraction after PEA. However, on an individual patient level, a decrease in shunt fraction was associated with a decrease in simplified four-strata risk score.

## 6. Limitation

This study’s sample size of 16 patients is relatively small but still considerable with respect to the rarity of the disease and the complexity of the measurements taken (e.g., pure oxygen breathing during RHC). This study’s findings need to be interpreted with caution due to the low sample size. In this study, only patients with operable CTEPH were included. Qs/Qt can only be calculated when breathing pure oxygen; it is not yet clear if shunt fractions change under different FiO_2_ and during exercise, though these would be difficult to assess [11].

## 7. Conclusions

We detected no significant improvement in Qs/Qt one year after pulmonary endarterectomy in patients with operable CTEPH, which may be explained by an inaccessible microvasculopathy. A decrease in Qs/Qt was associated with a decrease in simplified four-strata risk score.

## Figures and Tables

**Table 1:** Baseline characteristics (before PEA).

Number	16
Female, *n* (%)	6 (38)
Deceased, *n* (%)	0 (0)
Age, years	66 (55; 74)
Height, cm	174 (165; 180)
Weight, kg	81 (60; 91)
Body mass index, kg/m^2^	25.9 (22.1; 29.4)
Body surface area, m^2^	1.9 (1.7; 2.1)
6 min walking distance, m	512 (443; 570)
Oxygen saturation after 6 min walking test, %	88 (85; 93)
NT-proBNP, ng/L	409 (125; 968)
World Health Organization functional class	
WHO-FC II	8 (50)
WHO-FC III	7 (44)
WHO-FC IV	1 (6)
Pulmonary hypertension treatment before PEA	
Endothelin receptor antagonist	6 (38)
Phosphodiesterase-5 inhibitors	0 (0)
Soluble guanylate cyclase stimulator	7 (44)
Prostacyclin receptor agonist	0 (0)
Combination therapy	4 (25)
No therapy	8 (50)
Risk score	1.67 (1.08; 2.25)
Four-strata risk category	
Low	6 (38)
Intermediate low	7 (44)
Intermediate high	3 (19)
High	0 (0)
Pulmonary function tests, % predicted, *n* = 14	
FEV_1_	89.0 (79.5; 100.5)
FVC	91.5 (86.3; 105.5)
TLC	94.5 (85.8; 103.3)
DLCO	69.0 (63.8; 76.3)
Kco	80.5 (72.0; 89.3)

Data are presented as median (interquartile range) or number (%). NT-proBNP—*N*-terminal prohormone of brain natriuretic peptide; WHO-FC—World Health Organization functional class; FEV_1_—forced expiratory volume in the first second of expiration; FVC—forced vital capacity; TLC—total lung capacity; DLCO—diffusion capacity of carbon monoxide; K_co_—carbon monoxide transfer coefficient.

**Table 2:** Right-to-left shunt fraction (Qs/Qt) measured before and 1 year after pulmonary endarterectomy during right heart catheterization whilst breathing pure oxygen.

Fraction of Right-to-Left Shunt	Baseline	1 Year after PEA	*p*-Value
Shunt fraction, %	13.7 (10.0; 17.5)	13.0 (11.2; 15.6)	0.679
Shunt fraction > 5%	16 (100)	16 (100)	
Shunt fraction > 10%	12 (75)	14 (88)	
Patients with improved shunt by >10% of baseline value		7 (44)	
Patients that did not improve in shunt		9 (56)	

Data are presented as median (interquartile range) or number (%). PEA—pulmonary endarterectomy.

**Table 3:** Resting hemodynamics and blood gases during right heart catheterization whilst breathing ambient air at baseline and 1 year after pulmonary endarterectomy.

	Baseline	1 Year after PEA	
Resting Hemodynamics during RHC	Normoxia (FiO_2_ 0.21)	Normoxia (FiO_2_ 0.21)	*p*-Value
Oxygen saturation, %	93 (90; 96)	98 (95; 99)	0.002 *
Heart rate, min^−1^	70 (58; 78)	64 (60; 79)	0.641
Systolic blood pressure, mmHg	126 (119; 135)	141 (118; 146)	0.438
Diastolic blood pressure, mmHg	73 (66; 76)	77 (63; 86)	0.277
Mean pulmonary artery pressure, mmHg	38 (32; 41)	24 (18; 28)	<0.001 *
Pulmonary artery wedge pressure, mmHg	11 (10; 14)	11 (8; 12)	0.507
Right atrial pressure, mmHg	7 (6; 10)	6 (5; 8)	0.580
Cardiac index, L/min/m^2^	2.7 (2.1; 3.2)	2.7 (2.3; 3.0)	0.682
Pulmonary vascular resistance, WU	5.7 (4.0; 6.8)	2.5 (1.4; 3.8)	<0.001 *
Blood gases, arterial			
Hemoglobin, g/dL	15.1 (13.5; 15.7)	14.8 (13.9; 15.5)	0.972
Oxygen saturation, %	92 (88; 93)	94 (93; 95)	0.001 *
pH	7.44 (7.42; 7.47)	7.43 (7.40; 7.45)	0.423
Partial pressure of oxygen, kPa	8.5 (6.9; 9.3)	10.2 (9.4; 11.0)	0.002 *
Partial pressure of carbon dioxide, kPa	4.4 (3.9; 4.7)	4.6 (4.3; 5.2)	0.034 *
Blood gases, mixed venous			
Oxygen saturation, %	64 (57; 68)	69 (65; 72)	0.003 *
Partial pressure of oxygen, kPa	4.5 (4.1; 4.7)	5.0 (4.6; 5.2)	<0.001 *
Partial pressure of carbon dioxide, kPa	5.1 (4.6; 5.4)	5.4 (4.7; 5.5)	0.215
Oxygen content and delivery			
Oxygen delivery, mL/min	873 (680; 1029)	898 (808; 998)	0.352
Arterial content of oxygen, mL/dL	17.5 (16.5; 19.1)	18.7 (17.7; 19.5)	0.796
Mixed venous content of oxygen, mL/dL	12.6 (11.4; 13.6)	13.8 (12.5; 14.8)	0.034 *

Data are presented as median (interquartile range). FiO_2_—fraction of inspired oxygen; RHC—right heart catheterization; PEA—pulmonary endarterectomy. Statistically significant values are denoted with *.

**Table 4:** Exercise performance, WHO functional class, risk category, and laboratory parameters at baseline and 1 year after pulmonary endarterectomy.

	Baseline	1 Year after PEA	*p*-Value
WHO functional class			0.002 *
WHO-FC I	0 (0)	6 (38)	
WHO-FC II	8 (50)	10 (63)	
WHO-FC III	7 (44)	0 (0)	
WHO-FC IV	1 (6)	0 (0)	
6 min walking test (breathing ambient air)			
6MWD, m	512 (443; 570)	518 (462; 596)	0.221
SpO_2_ at end exercise, %	88 (85; 93)	93 (87; 96)	0.319
NT-proBNP, ng/L	409 (125; 968)	206 (94; 437)	0.233
Four-strata risk category			
Low	6 (38)	12 (75)	
Intermediate low	7 (44)	4 (25)	
Intermediate high	3 (19)	0 (0)	
High	0 (0)	0 (0)	
Risk score	1.67 (1.08; 2.25)	1.00 (1.00; 1.59)	0.015 *

Data are presented as median (interquartile range) and number (%). PEA—pulmonary endarterectomy; WHO—World Health Organization; 6MWD—6 min walking distance; SpO_2_—oxygen saturation; NT-proBNP—*N*-terminal prohormone of brain natriuretic peptide. Statistically significant values are denoted with *.

**Table 5:** Changes in main hemodynamic parameters, 6 min walk test, WHO functional class, and NT-proBNP stratified for patients with improvements and no improvements in shunt fraction.

		Median (IQR)			Test Statistics
	*n*	Baseline	*n*	1 Year after PEA	*p*-Value
Improvement in shunt fraction					
Oxygen saturation, %	7	92 (90; 97)	7	97 (95; 99)	0.028 *
Mean pulmonary artery pressure, mmHg	7	37 (29; 48)	7	24 (19; 28)	0.018 *
Pulmonary vascular resistance, WU	7	6.5 (3.4; 6.8)	7	2.8 (1.5; 3.8)	0.043 *
Cardiac index, L/min/m^2^	7	2.8 (2.0; 3.2)	7	2.4 (2.2; 3.3)	0.610
WHO functional class	7	3 (2; 3)	7	2 (1; 2)	0.038 *
NT-proBNP, ng/L	7	436 (100; 976)	7	125 (76; 349)	0.115
6 min walking distance, m	7	552 (420; 618)	6	557 (511; 602)	0.173
SpO_2_ at end 6MWD, %	7	88 (87; 93)	6	93 (88; 97)	0.916
Risk score	7	1.67 (1.0; 2.33)	7	1.00 (1.00; 1.33)	0.042 *
No improvement in shunt fraction					
Oxygen saturation, %	9	94 (90; 96)	9	98 (95; 99)	0.021 *
Mean pulmonary artery pressure, mmHg	9	38 (33; 41)		23 (17; 29)	0.008 *
Pulmonary vascular resistance, WU	9	4.2 (4.1; 7.9)	9	2.4 (1.3; 3.2)	0.008 *
Cardiac index, L/min/m^2^	9	2.7 (2.1; 2.9)	9	2.7 (2.6; 2.9)	0.174
WHO functional class	9	2 (2; 3)	9	2 (1; 2)	0.023 *
NT-proBNP, ng/L	9	381 (141; 1058)	9	220 (170; 611)	0.678
6 min walking distance, m	9	508 (446; 566)	9	490 (387; 630)	0.674
SpO_2_ at end 6MWT, %	9	86 (81; 93)	9	93 (85; 96)	0.286
Risk score	9	1.67 (1.17; 2.34)	9	1.00 (1.00; 1.67)	0.118

Data are presented as median (interquartile range). WHO—World Health Organization; NT-proBNP—*N*-terminal prohormone of brain natriuretic peptide; SpO_2_—oxygen saturation; 6MWD—6 min walking distance. Statistically significant values are denoted with *.

**Table 6:** Changes in hemodynamic parameters, 6 min walk test, functional class, and NT-proBNP from baseline to 1 year after pulmonary endarterectomy overall and stratified for patients with and without improvements in shunt fraction.

Variables	Overall Sample	Improvement in Shunt Fraction	No Improvement in Shunt Fraction	*p*-Value
	(*n* = 16)	(*n* = 7)	(*n* = 9)	
∆ Mean pulmonary artery pressure, mmHg	−14 (−21; −7)	−14 (−21; −4)	−14 (−21; −9)	0.837
∆ Pulmonary vascular resistance, WU	−2.7 (−4.9; −0.7)	−2.9 (−5.0; −0.6)	−2.4 (−6.5; −0.9)	0.681
∆ 6MWD, m	28 (−21; 106)	36 (−13; 145)	0 (−61; 107)	0.689
∆ NT-proBNP, ng/L	−19 (−704; 60)	−19 (−692; 0)	−1 (−733; 273)	0.681
WHO functional class				1.000
Improvement (*n*, %)	11 (69)	5 (71)	6 (67)	
No improvement (*n*, %)	5 (31)	2 (29)	3 (33)	
NT-proBNP, ng/L				0.633
Improvement (*n*, %)	10 (63)	5 (71)	5 (56)	
No improvement (*n*, %)	6 (38)	2 (29)	4 (44)	
6 min walking distance, m				0.608
Improvement (*n*, %)	8 (53)	4 (67)	4 (44)	
No improvement (*n*, %)	7 (47)	2 (33)	5 (56)	
SpO_2_ end 6MWD, %				0.608
Improvement (*n*, %)	7 (47)	2 (33)	5 (56)	
No improvement (*n*, %)	8 (53)	4 (67)	4 (44)	
Risk score				1.000
Improvement (*n*, %)	11 (69)	5 (71)	6 (67)	
No improvement (*n*, %)	5 (31)	2 (29)	3 (33)	
Four-strata risk category				0.633
Improvement (*n*, %)	10 (63)	5 (71)	5 (56)	
No improvement (*n*, %)	6 (38)	2 (29)	4 (44)	
Residual PH				1.000
Yes (*n*, %)	8 (50)	4 (57)	4 (44)	
No (*n*, %)	8 (50)	3 (43)	5 (56)	
Pulmonary hypertension treatment before PEA				1.000
Yes (*n*, %)	8 (50)	3 (43)	5 (56)	
No (*n*, %)	8 (50)	4 (57)	4 (44)	
Pulmonary hypertension treatment after PEA				1.000
Yes (*n*, %)	5 (31)	2 (29)	3 (33)	
No (*n*, %)	11 (69)	5 (71)	6 (67)	

Data are presented as median (interquartile range) or number (%). 6MWD—6 min walking distance; NT-proBNP—N-terminal prohormone of brain natriuretic peptide; WHO—World Health Organization; SpO_2_—oxygen saturation; Residual PH—residual pulmonary hypertension.

## Data Availability

The dataset used for this study can be made available by the corresponding author upon request.

## Abbreviations

BMI	Body mass index
BP	Blood pressure
BPA	Balloon pulmonary angioplasty
BSA	Body surface area
CI	Cardiac index
CO	Cardiac output
CTEPH	Chronic thromboembolic pulmonary hypertension
ERS	European Respiratory Society
ESC	European Society of Cardiology
FiO_2_	Fraction of inspired oxygen
Hb	Hemoglobin
HR	Heart rate
mPAP	Mean pulmonary artery pressure
NT-proBNP	*N*-terminal prohormone of brain natriuretic peptide
PAH	Pulmonary arterial hypertension
PAWP	Pulmonary artery wedge pressure
PEA	Pulmonary endarterectomy
PH	Pulmonary hypertension
PVR	Pulmonary vascular resistance
Qs/Qt	Fraction of right-to-left shunt
RHC	Right heart catheterization
WHO	World Health Organization
WU	Wood unit
6MWD	6 min walking distance

## Appendix A

**Table A1:** Hemodynamics and blood gases during right heart catheterization whilst breathing pure oxygen at baseline and 1 year after pulmonary endarterectomy.

	Baseline	1 Year after PEA
Resting Hemodynamics during RHC	Hyperoxia (FiO_2_ 1.0)	Hyperoxia (FiO_2_ 1.0)
Oxygen saturation, %	100 (99; 100)	100 (100; 100)
Heart rate, min^−1^	63 (54; 71)	65 (59; 75)
Systolic blood pressure, mmHg	126 (118; 132)	136 (120; 151)
Diastolic blood pressure, mmHg	71 (63; 79)	81 (67; 85)
Mean pulmonary artery pressure, mmHg	33 (27; 39)	20 (17; 24)
Pulmonary artery wedge pressure, mmHg	11 (8.5; 14)	10 (8; 12)
Right atrial pressure, mmHg	7 (4; 7)	5 (4; 8)
Cardiac index, L/min/m^2^	2.3 (1.9; 2.7)	2.6 (2.3; 3.2)
Pulmonary vascular resistance, WU	4.7 (3.5; 6.7)	2.0 (1.1; 3.0)
Blood gases, arterial		
Hemoglobin, g/dL	14.6 (13.7; 15.7)	15.0 (13.8; 15.6)
Oxygen saturation, %	98 (98; 99)	98 (98; 99)
pH	7.44 (7.43; 7.50)	7.45 (7.41; 7.47)
Partial pressure of oxygen, kPa	62.8 (59.2; 67.9)	69.2 (56.0; 71.8)
Partial pressure of carbon dioxide, kPa	4.1 (3.6; 4.6)	4.3 (3.9; 4.9)
Blood gases, mixed venous		
Oxygen saturation, %	78 (74; 83)	80 (77; 82)
Partial pressure of oxygen, kPa	5.8 (5.0; 6.6)	6.1 (5.5; 6.7)
Partial pressure of carbon dioxide, kPa	4.9 (4.4; 5.5)	4.9 (4.5; 5.7)
Oxygen content and delivery		
Oxygen delivery, mL/min	904 (678; 995)	962 (873; 1057)
Pulmonary capillary content of oxygen, mL/dL	19.8 (18.7; 21.3)	20.3 (18.7; 21.1)
Arterial content of oxygen, mL/dL	19.5 (18.2; 20.9)	19.8 (18.3; 20.7)
Mixed venous content of oxygen, mL/dL	15.6 (14.4; 16.1)	15.8 (15.0; 16.4)

FiO_2_—fraction of inspired oxygen; PEA—pulmonary endarterectomy; RHC—right heart catheterization.
